# Supplementary material for: Development and testing of the ‘Culture of Care Barometer’ (CoCB) in healthcare organisations: a mixed methods study
Source: BMJ Open. 2017 Aug 18;7(8):e016677. doi: 10.1136/bmjopen-2017-016677 (PMC5629717; doi:10.1136/bmjopen-2017-016677)
Supplement: Author's manuscript [file bmjopen-2017-016677.draft_revisions.pdf]

BMJ Open

The Culture of Care Barometer: Development and testing of a tool for use in healthcare settings.

Journal:	*BMJ Open*
Manuscript ID	bmjopen-2017-016677
Article Type:	Research
Date Submitted by the Author:	01-Mar-2017
Complete List of Authors:	Rafferty, Anne Marie; King's College London, Florence Nightingale School of Nursing and Midwifery<br>Philippou, Julia; King's College London, Nursing & Midwifery<br>Fitzpatrick, Joanne; King's College London, Nursing & Midwifery<br>Pike, Geoff; Employment Research<br>Ball, Jane; National Institute for Health Research Collaboration for Leadership in Applied Health Research and Care (Wessex),University of Southampton, Centre for Innovation and Leadership in Health Sciences
**Primary Subject Heading**:	Nursing
Secondary Subject Heading:	Health services research
Keywords:	Culture of Care, tool development, Organisational development < HEALTH SERVICES ADMINISTRATION & MANAGEMENT, quality in healthcare, Human resource management < HEALTH SERVICES ADMINISTRATION & MANAGEMENT

SCHOLARONE™
Manuscripts

Title page

The Culture of Care Barometer: Development and testing of a tool for use in healthcare settings.

Full name, postal address, e-mail and telephone number of the corresponding author:

Professor Anne Marie Rafferty

57 Waterloo Road

London SE1 8WA

Email: anne_marie.rafferty@kcl.ac.uk

Tel: 02078483012/3984

Full name, department, institution, city and country of all co-authors:

Professor Anne Marie Rafferty, Florence Nightingale Faculty of Nursing and Midwifery, King's College London, London UK.

Dr Julia Philippou, Florence Nightingale Faculty of Nursing and Midwifery, King's College London, London UK.

Dr Joanne M. Fitzpatrick, Florence Nightingale Faculty of Nursing and Midwifery, King's College London, London UK.

Geoff Pike, Employment Research, Hove, UK.

Jane Ball, Faculty of Health Sciences, University of Southampton, Southampton UK & Karolinska Institute, Stockholm, Sweden.

Word count, excluding title page, abstract, references, figures and tables: 3983 (Max 4000)

For peer review only

For peer review only - http://bmjopen.bmj.com/site/about/guidelines.xhtml

Abstract (269 Words)

Objective: Concerns about care quality have prompted calls to create workplace cultures conducive to high quality, safe and compassionate care and provide a supportive environment in which staff can operate effectively. How healthcare organisations assess their culture of care is an important first step in creating such cultures. This paper reports on the development and validation of a tool designed to assess perceptions of a caring culture among healthcare workers preliminary to culture change.

Design/Setting/Participants: An exploratory mixed methods study designed to develop and test the validity of a tool to measure 'culture of care' through focus groups and questionnaires. Questionnaire development was facilitated through; a literature review, experts generating items of interest and focus group discussions with healthcare staff across specialities, roles and seniority within three types of public healthcare organisations in the UK. The tool was designed to be multi-professional and pilot tested with a sample of 467 nurses and healthcare support workers in acute care and then validated with a sample of 1698 staff working across acute, mental health and community services in England. Exploratory factor analysis was used to identify dimensions underlying the Barometer.

Results: Psychometric testing resulted in the development of a 30-item questionnaire linked to four domains with retained items loading to four factors: organizational values (α=0.93, valid n=1568, M= 3.7); team support (α=0.93, valid n=1557, M=3.2); relationships with colleagues (α=0.84, valid n=1617, M=4.0) and job constraints (α=0.70, valid n=1616, M=3.3).

Conclusions: The study developed a valid and reliable instrument with which to gauge the different attributes of care culture perceived by healthcare staff with potential for organisational benchmarking.

Strengths and limitations of the study

- This study explored the reliability, content validity and factorial structure of the Culture of Care Barometer (CoCB) tool.
- The CoCB tool has potential use in healthcare settings and research and can be used at macro, meso and micro level within organizations to measure the culture of care.
- The dual emphasis of the tool on the dialogic and diagnostic aspects of organisational development theory allows organisations to assess their culture of care and stimulate dialogue and reflection on questions of culture and what is perceived to be of particular value within teams.
- Early indications demonstrate that the tool has good face validity with regard to key factors associated with a culture of care: Trust level values, team support, relationships with colleagues and job constraints.
- The CoCB requires further validation to ascertain the concurrent and predictive validity and to evaluate the usefulness and acceptability of the tool across different care settings and with a wider population.

For peer review only - http://bmjopen.bmj.com/site/about/guidelines.xhtml

Main text

BACKGROUND

The importance of culture in providing high quality and safe care to patients has been emphasized in many investigations of failings in healthcare systems both nationally [1, 2] and internationally [3]. Healthcare organizations have begun to look critically at ways that can improve their culture and consequently the care provided to patients [4]. In the United Kingdom (UK), the Care Quality Commission, has drawn attention to “cultures of care that are too often ‘task-based’ when they should be person-centred, and where the unacceptable become the norm” [5](pg.5) noting variation in cultures within organizations reflecting leadership and management failings [6]. Learning from high profile failures in care delivery indicates that quality and culture are not uniform within, let alone across, organisations [6, 7]. This was evident in the description and analysis of events (and the context to those events) at Mid Staffordshire National Health Service (NHS) Trust, described by the Robert Francis Inquiry [1]. Pockets of excellence can coexist alongside the worst examples of care failures [6]; lack of consistency in care culture impedes the spread of good practice across organisations [1, 8]. Establishing cultures that will allow healthcare organisations to achieve the ultimate goal of providing high quality care has therefore become a major policy concern.

Evidence suggests that major failures are frequently not brought to light by the systems for quality assurance or improvement that are part of most healthcare organizations in developed countries - such as incidence reporting, mortality and morbidity reviews, inspections, accreditations, clinical profiling and risk and claim management [3]. Since these cultural attributes are not picked up in the measures of quality and performance currently in use; metrics fail to capture the meaning and reality of a culture of care for patients or staff. Moreover, research in the UK demonstrates that the wellbeing of staff is closely linked to the wellbeing of patients, and staff engagement is a key predictor of a wide range of outcomes in healthcare organizations [9].

One of the first questions organization have to consider in trying to establish such care cultures is how to assess the organizational culture. The first difficulty in this is that the concept of culture is broad and multi-faceted [10]. While culture as a concept is widely used, the term itself has been described as an ‘indescribable mist’ [10]. Conceptual debates over how culture is defined continue, and consequently impact how it is studied [10]. Much of the literature in healthcare favours the concept of culture as shared beliefs, norms and routines through which a society can be interpreted and understood [11]. With this definition in mind our focus was on understanding the culture of care, as a subset of organisational culture, and help organizations gauge the different attributes of caring environments.

From an organisational development (OD) perspective - the practical application of culture assessment tools speaks to the diagnostic premises of OD theory and practices. Such tools ascertain the strengths and weaknesses of organizations and help them prescribe interventions or ‘treatments’ of change based on an objective diagnosis from the data collected [12]. Within healthcare there is a plethora of well-known instruments for measuring the culture of organizations and ‘patient safety’ culture [13]. In a national survey of healthcare organizations in the UK to identify the culture assessment tools that are used within the English NHS, concluded that while organizations are increasingly using culture assessment instruments these focus primarily on the assessment of safety culture rather than perspectives of quality [13]. Moreover, while the centrality of patients’ experiences, of safety, caring and supportive cultures, in such tools is well evidenced, a large research programme examining culture and behaviour in the English NHS concluded that

For peer review only - http://bmjopen.bmj.com/site/about/guidelines.xhtml

creating caring cultures where staff can feel supported, respected, valued and engaged are equally important for providing high quality care [7]. Therefore, it is argued that achieving the optimal care culture is only possible in organizations where staff feel valued, respected and supported, and when relationships are good between managers, staff, teams, departments and across institutional boundaries [7, 9, 10]. An initial analysis of the literature revealed a lack of instruments for measuring 'care cultures' from the perspective of service providers as distinct from organizational culture or patient safety culture [14].

Diagnostic OD entails a problem-based approach where organisations are considered to be problematic and need fixing [15]. This approach to data gathering has been described as 'problem-sensing' [7] as it actively seeks out weaknesses in organizational systems. This can result in organizational members being wary as they may feel that the main purpose of data collection is to attribute blame; ultimately this can inhibit or make members more resistant to change [7, 12]. One of the most sensitive messages coming out from Mid-Staffordshire Inquiry was how staff suffered as a result of raising concerns [1]. This has led to a review of the way NHS organizations deal with concerns raised by NHS staff, advocating for a culture of safety and learning in which staff feel safe to raise concerns and these conversations take place as part of everyday practice without fear of blame or recrimination [15]. Previous research identified that extant culture assessment tools failed to address culture attributes that promote the development of a blame-free environment [13].

In contrast to diagnostic OD approaches, dialogic approaches are primarily concerned with 'meaning-making' [12]. Building a more dialogical approach to OD [12] therefore could encourage reflection and stimulate discussion about the culture of an organization and how 'care givers' express and create meaning in their performance of care [14]. Given the gaps in current culture measurement tools we aimed to develop a tool that could act as a 'diagnostic' measurement to help organisations assess the culture of care but also as a 'dialogic' tool designed to prompt reflection on the underlying issues involved in creating a caring culture. The current paper presents the development and testing of the Culture of Care Barometer (CoCB) as a tool that has the potential to serve these purposes.

METHODS

We followed well-recognized and comprehensive approaches for instrument development and testing e.g. Hinkin's framework for scale development [17, 18, 19], and pursued a variety of data collection and analysis methods to operationalize the elements that are important in creating caring cultures and to ensure the reliability of the tool [20]. A detailed account of the process of developing and testing the CoCB is provided below. Figure one provides an overview and graphical representation of this process.

For peer review only - http://bmjopen.bmj.com/site/about/guidelines.xhtml

Insert Figure 1 Here

Item generation

We used a mixed method approach in the creation of items to assess the culture of care construct. Our processes involved both inductive and deductive approaches to generate items [17, 18]. Initially an inductive approach was used where an expert panel of six healthcare leaders developed a prototype questionnaire by generating items and domains they considered important in improving patient care. The expert panel consisted individuals with extensive experience and expertise in regulation, leadership, healthcare-delivery, management, policy and research within the English NHS, including the use of tools used in inspection regimes. This process was complemented by a deductive approach involving a comprehensive literature review in four major healthcare related electronic database resources (CINAHL, EMBASE, MEDLINE, and Web of Science; from 1945 to 2015) using key terms such as 'organisational culture' 'assessment tools', 'healthcare settings' and 'quality of care'. Through the literature review key studies [e.g. [7, 20, 21, 22, 23]] and previous comprehensive reviews on validated scales measuring organisational culture [e.g. [14, 13, 24, 25]] within healthcare were extracted and together, these two complementary processes helped us create a comprehensive pool of candidate items that could be used to measure the concept of the culture of care.

Content adequacy assessment

A comprehensive approach was also followed at the stage of content adequacy to test the conceptual consistency of the items and assure content validity of the questionnaire. In order to achieve this we undertook six focus groups with a cross section of healthcare staff (N=34) of different levels of seniority and from different settings e.g. both in-patient and out-patient. The aim of these focus groups was to explore perceptions of terminology and cognitively test items and their meaning to enable a 'co-creation approach' to the development of the tool with frontline staff. This was envisaged as a process that would allow the tool development stage to address some limitations reported in the literature in terms of developing tools that are 'fit for purpose' in the NHS context. The process also allowed us to explore staff ideas of what constitutes a 'good culture of care' and key 'signs and symptoms' of an organisation that has a good or poor culture of care and identify any additional items relevant to the concept. At the same time we were able to test the appropriateness, comprehension and clarity of items, and ease and acceptability of instructions and format of the initial questionnaire [19].

At this stage an initial tool of 37 items clustered under four domains was developed and a five point scale, ranging from not at all, to fully agree, was used to record participants' agreement with the items. The first domain comprised six items that explored issues surrounding resources and quality of care, facilities and equipment, staffing levels and views of workplace in terms of safety and quality. It included the Friends and Family Test as a reference item [26] and a question about action required to improve resources. The second domain comprised 10 items relating to management and support. Ten items in the third domain addressed development, staff involvement in decision making and overall culture in the organisation. The fourth domain included 11 items about staff meetings, teamwork and feedback and willingness of the organisation to learn from issues raised as well as incidents. For each domain a question was included about how much influence participants

For peer review only - http://bmjopen.bmj.com/site/about/guidelines.xhtml

had to improve things, rated on a five point sale (from 'none', to 'a lot'). The initial tool is provided in full in the study's report [27].

The tool was then pilot tested in an acute NHS organization with a sample of registered nurses and midwives and healthcare support workers (n=467, 24% response rate). The aim of this pilot testing was to examine how well the tool performed and test the face validity and internal reliability of the items before wider testing. An initial factor analysis was conducted to reduce the number of items and further refine the tool. Results from this pilot study are reported in the study's report [27]. The items identified through this analysis were explored by the research team using an index-card-sorting exercise [28]. This involved asking the members of the research team to sort the items into categories. The items were printed separately on a small index card each member of the team sorting the cards into groups. Each then described what they saw as the common theme relating to the cards in each group. Through this analysis and process, 30 items were retained in the questionnaire representing the concept of culture of care and these were classified under seven categories: engagement, empowerment, management and leadership, values, roles, resources and team. Before administering the final questionnaire to a wider sample a small number of NHS staff reviewed the items and the overall questionnaire to identify whether this provided an accurate representation of the overall culture in their workplace. The revised version of the tool is available in full in the study report [27].

Questionnaire administration

The retained items and revised tool was administered to a wider sample with the objective of examining how well the tool performed and how well the remaining items confirmed expectations of the psychometric properties of the tool. Below we present the main procedures we followed during this stage of development and testing the CoCB tool.

Administration of the questionnaire was undertaken in two further healthcare organisations. One was a mixed mental health care provider in London with community care and in-patient beds and the other was a predominately community healthcare organisation in a mixed urban/rural setting in the South of England. A total sample approach was adopted and an electronic version of the questionnaire was send to the two organisations for distribution. We also forwarded 1500 paper copy questionnaires for each organisation to distribute to participants with less access to the electronic version. Questionnaires were distributed in June 2014 and data collection lasted for about eight weeks.

Factor analysis and internal consistency assessment of the tool

Data were coded and entered into a Microsoft Excel spreadsheet before exported and analysed through IBM SPSS v21 statistical software. The demographic and employment characteristics of the participants were analysed descriptively and presented as numbers and percentages. A principle component of exploratory factor analysis was undertaken to identify whether the correlations between a groups of observed items originated from one or more latent variables/factors in the data. Internal consistency was tested using Cronbach's coefficient α. An Alpha reliability score above 0.7 is considered good [29, 28] therefore the target level of reliability was set at 0.70.

RESULTS

For peer review only - http://bmjopen.bmj.com/site/about/guidelines.xhtml

Sample characteristics

A total of 1705 staff working across mental health and community services in both clinical and non-clinical roles returned the questionnaires organization 1 (n=700, 25% response rate); organization 2 (n=1005, 24% response rate), of which 1698 were found complete and eligible for statistical analysis. Table one summarise the main profile and employment characteristics of participants. Overall four-in-five (82%, n=1237) respondents were female, with a large proportion of participants indicating being age 40 or older (Table 1). Almost all sample participants (93%, n=1459) spoke English as their first or main language. There were no difference in the demographic variable by organization. Two-thirds of staff across the two organisations worked full-time (68%, n=1074). By staff group there was a similar responded profile within the two organizations and overall a larger proportion of nurses responded to the questionnaire compared to other group of staff.

Table 1: Characteristics of participants

	Percentage (n)		
	**Organization 1 (n=700)**	**Organization 2 (n=1005)**	**Overall (n=1705)**
**Female**	**75**	**87**	**82**
**English as a first or main language**	**86**	**98**	**93**
**Age groups**			
<29	8	8	8
30-39	17	17	17
40-49	33	32	32
50-59	32	35	34
60 plus	9	8	8
**Staff group**			
Registered Nurses and Health Visiting Staff	30	34	32
Healthcare Assistant/Support Worker	13	5	8
Allied Health Professionals	25	25	25
Estates and facilities	2	4	3
Doctor/Dentist	5	3	4
Administrative and Clerical Staff	15	15	15
Central Functions and Corporate Services	4	6	5
Other	4	6	5
**Work setting**			
Community	36	45	41
Clinics/Outpatient Departments	13	14	14
Wards/Inpatient Units	27	7	15
Office	16	27	23
Other	9	7	7
**Employment status**			
Full-time	76	64	68
Part-time	25	36	32

Structure of the tool

A factor analysis was performed on the 30 items to identify patterns of loading and extract underlying factors. Through this analysis four factors were identified. The composition of the factors was based on aggregating the scores for each item to create a single score for each factor and

For peer review only - http://bmjopen.bmj.com/site/about/guidelines.xhtml
For peer review only

dividing this figure by the number of item in the CoCB tool to provide a meaningful average score. The relevant loading of items for each of the four factors is presented in Table 2.

The 12 items of the first sub scale were predominately related with macro level elements within organizations that influence culture such as valuing employees, good communication within the organization and visible leadership at top level. Therefore, this subscale was consider addressing wider 'organizational values'. Further 11 items were loading to the second factor labelled as Team support. Items loading to this subscale were concerned with the 'meso level' of organizations structure and included elements that described primarily team support relationships and management and development of employees within organizations. The remaining seven items concerned aspects of every day work at micro level within organizations and these were loading into two factors. One sub-scale was mainly associated with four items describing social elements of work such as respect and social support between co-workers and the final three items were concerned with the ability of employees to do their job within the limits of time and resources available to them (Factor 4).

Table 2: Factor analysis and loading of items

Subscales and items	Loading
**Factor 1: Organizational Values (Macro Level)**	
The Trust listens to staff views	0.84
The Trust has a positive culture	0.77
There is strong leadership at the highest level in the Trust	0.75
I am able to influence how things are done in the Trust	0.74
I would recommend this Trust as a good place to work	0.70
I feel well informed about what is happening in the Trust	0.70
Staff successes are celebrated by the Trust	0.70
The Trust values the service we provide	0.69
Trust managers know how things really are	0.68
I am proud to work in this Trust	0.65
A positive culture is visible where I work	0.50
I get the training and development I need	0.40
**Factor 2: Team Support (Meso Level)**	
I feel well supported by my line manager	0.87
My line manager treats me with respect	0.84
My line manager gives me constructive feedback	0.83
My concerns are taken seriously by my line manager	0.81
I feel part of a well-managed team	0.60
I am kept well informed about what is going on in our team	0.52
I feel supported to develop my potential	0.50
I know who my line manager is	0.45
I am able to influence the way things are done in my team	0.44
I feel able to ask for help when I need it	0.44
Unacceptable behaviour is consistently tackled	0.40
**Factor 3: Relationships with colleagues (Micro Level)**	
The people I work with are friendly	0.81
When things get difficult, I can rely on my colleagues	0.79
I feel respected by my co-workers	0.76
I have positive role models where I work	0.56
**Factor 4: Job constrains (Micro Level)**	
I have sufficient time to do my job well	0.79
I have the resources I need to do a good job	0.72

For peer review only - http://bmjopen.bmj.com/site/about/guidelines.xhtml

I know exactly what is expected of me in my job	0.41

Reliability analyses were performed on each factor to identify how items loading to factors are considered a positive endorsement of the sub-scale. The results of these analyses are presented in table 3. Cronbach's α for the macro level (Factor 1: Organizational values) and meso level scales (Factor 2: Team support) were very high both at 0.93. Cronbach's α for the micro level factors were: 0.84 (Factor 3: Relationships with colleagues) and 0.70 (Factor 4: Job constrains) accordingly.

Table 3: Scale characteristics (Cronbach's coefficient α reliability analysis)

	**Factor 1** Organizational values (Macro Level)	**Factor 2** Team support (Meso Level)	**Factor 3** Relationship with Colleagues (Micro Level)	**Factor 4** Job constrains (Micro Level)
Total Items loading	12	11	4	3
Alpha reliability	0.93	0.93	0.84	0.70
Number of valid responses	1568	1557	1617	1616
Mean score	3.7	3.2	4.0	3.3

Usefulness and added value of the tool to healthcare organizations

The results from the CoCB were presented to the healthcare organisations through two independent reports detailing the findings from testing the tool in each organisation. Following the presentation of the results we invited key individuals from each organization (n=5) to a follow up discussions to receive feedback on the usefulness of the tool and explore whether the tool meets dialogic and diagnostic premises of organizational development [12]. These individuals held strategic leadership positions within the two organizations with responsibilities of overseeing workforce and culture initiatives. These sessions were audio recorded and analysed thematically.

From a diagnostic perspective the consensus overall was that the CoCB tool resonated with other instruments used in the organisations, adding 'colour and depth' to them.

> *"I think it is a much richer type of feedback than we get from the staff survey. We liked the logic and flow and could appreciate the sense of questions." (Organizational Development Manager, Organization 2)*

> *"…you get these action plans that come out of the staff surveys, but the detail is not the depth information that we had with this, so this is for more….enables you to think more about why, then, and question, as oppose to, 'oh, right, we've got to do something on that area." (Senior Manager, Organization 1)*

The brevity of the tool, the fact that it was easy to complete and was perceived as targeting the right domains was appreciated by staff. Moreover, from a diagnostic perspective the tool was perceived by participants as useful in providing a reference point for them to gauge where they were on a cultural spectrum or journey.

For peer review only - http://bmjopen.bmj.com/site/about/guidelines.xhtml

From a dialogical perspective the fundamental value of the CoCB tool was reflected in the belief that 'culture changes by talking about it' and the Barometer helped to surface issues for discussion. Data from the COCB were seen as helpful in drilling into further detail or using it as a prompt for a 'quality conversation' for instance, with smaller, discrete groups, teams or where it was felt things were not quite right or when organisations felt the need to gauge the impact of changes they had made.

> *'Culture …does not change overnight and the fact that it is [the CoCB tool] a prompt to reflect upon has been I think really powerful aspect of the tool.'(Senior Executive, Organization 2).*

Finally, the commentary element of the tool was identified as providing a rich source of intelligence in helping to unpack notions of culture:

> *"trying to understand what it is that matters to staff and what they feel about the place that they work in" (Workforce Transformation Lead, Organization 2).*

It was also regarded as helpful in picking up on contradictions that might exist in organisations, as one senior manager observed:

> *"where you've got high scores for 'my manager treats me with respect', but then, 'Oh, I don't think my manager understands what the real world is like' (Leadership Project Manager, Organization 1).*

Richer feedback via the CoCB in comparison to the staff survey helped to tease out the contradictions and take the quality of conversation at team level to the next stage.

DISCUSSION

Enabling the workforce to put the right things in place for patients is key to improving NHS performance in terms of quality and safety [30] and this is the underlying principle of the CoCB as a tool. The challenge all organisations face is that there is not a one-size-fits-all solution as each individual is unique and will react differently to the challenges and values of an organisation. The CoCB can provide feedback from staff to enhance understanding of the factors that contribute to a culture lacking in care and safety. The CoCB appeared to perform well in meeting the gap in the literature which suggests that there is a need for tools to assess culture with a focus on formative diagnostic purposes to support reflexive practice [13]. The focus on the carers' views helps to comprehend the intersection of individual and organisational factors which distinguish the CoCB from other tools that prioritise either macro or micro levels. Moreover, using a dialogic approach to organisational development we created a tool that can be a resource to facilitate the involvement of frontline staff, at different levels and with different roles, in culture change initiatives.

The CoCB tool possess several advantages over existing tools. During the development and testing of the tool we addressed concerns reported in the literature and from participants about the need for tools that are 'fit for purpose' [13] and are not onerous and time consuming to complete. One of the major strengths of this study was the co-creation of the tool in collaboration with frontline staff providing care within NHS organisations. Three main sources of data information were used to create the content of the tool including a prototype tool developed by an expert panel in this field, a comprehensive literature review of the concept of culture of care and other tools used within healthcare plus and input from interviews from staff working in healthcare organisations. Feedback

For peer review only - http://bmjopen.bmj.com/site/about/guidelines.xhtml

from participants indicated that the data collected via the CoCB could provide extra intelligence about the jigsaw of what is happening within organisations. Participants indicated that the tool could enhance or complement current data collection methods used in hospital settings such as the Friends and Family Test and the Staff Survey to identify concerns about poor care. In this way providing a more comprehensive picture of patient and staff satisfaction with services provided. In addition, participants' spoke of the struggle to make meaningful changes based on Staff Survey feedback alone and they shared that data collected via the CoCB alerted them to important contextual feedback and factors that in some cases were more useful in planning and developing action plans.

While the CoCB is a reliable tool and does seem to fill a niche for identifying and understanding some of the social processes at work within an organisation, we recognise that not all factors contributing to developing a caring culture may be included in the tool. We also acknowledge that not all the items may be relevant to all healthcare contexts and we recommend that testing and adaptation of these items may be necessary in future validations. Therefore, practitioners should use the CoCB in conjunction with other tools that can help organisations achieve culture change. Moreover, the sample of this testing was predominately nurse led, which may be a direct result of the proportion of nurse personnel working in the healthcare environments or may be reflecting the high media profile of nursing post Francis prompting engagement with culture of care issues. However, cultures are co-created by all members of an organisation and this means that everyone is responsible for the welfare of the organisation as a whole. Further work is needed to test the reliability and validity of the questionnaire as well as engagement and uptake of the tool from other professional groups.

Measuring and monitoring culture is a recurrent challenge in healthcare, the CoCB could act as a diagnostic, and practical tool for organisations to embrace as a first step in improvement work as well as a means of monitoring change over time. The tool has potential use in clinical practice and research. It is easy to administer, can be completed and analysed quickly providing timely feedback that can be used by organisations to identify areas of strength and weakness and help with the planning of continuous quality improvements or culture change initiatives that hospitals are undertaking. The wider applicability of the tool needs to be explored in future studies and reach out to groups that may not have a direct role in care provision but who nevertheless are an important part of the organisation and thus their feedback is important as culture is every one's business [23]. At the time of writing a digital version of the tool is being developed. Further research should extend the current efforts and refine and evaluate the impact of the CoCB as necessary in order in order to develop interventions that can improve the culture of care in healthcare environments.

Twitter Follow Anne Marie Rafferty at @annemarieraffer, Julia Philippou at @julia_philippou and Jane Ball at @JaneEBall

Acknowledgments

We would like to thank all those who contributed to this work, particularly the staff who took part in in the survey and those who engaged in the focus groups. We also acknowledge the role of the Passionate About Care Today 'PACT' group (Baroness Audrey Emerton, Professor Dame Elizabeth Fradd, Professor Tricia Hart, Sir Stephen Moss, Flo Panel Coates, Professor Anne Marie Rafferty) in creating the prototype Barometer upon which this work is based and for their time and insights throughout the subsequent development of the tool. We are grateful to the support and insights

For peer review only - http://bmjopen.bmj.com/site/about/guidelines.xhtml

provided by the Steering Group for the project: Caroline Alexander, Bronagh Scott, Flo Panel Coates, Yvonne Coghill, Morvia Gooden; Sylvia Tang, Virginia Minogue, Paul Taylor and Nigel Charlesworth Hana Wild Funding to support the study was provided by NHS England.

Contributors

AMR was the principal investigator and with JB conceived and designed the study. AMR, JP, JF, GP and JB all contributed to the collection, analysis, or interpretation of data for the work. AMR and JP developed the first draft of the paper and all the authors contributed to different sections and revised the paper critically for intellectual content. All authors gave final approval of the version to be publishes and agree to be accountable for all aspects of the work in ensuring that question related to accuracy or integrity of any part of the work are appropriately investigated and resolved.

Funding

This work was supported by funding received from NHS London and NHS Engalnd.The indepentend research reported in this paper formed part of the Compassion in Practice, national strategy for nursing, midwifery and care staff, led by Jane Cummings the Chief Nursing Officer for NHS England, under action area four ‘Building and Strengthening Leadership-leading with Compassion’.

Competing interests

None declared.

Data sharing statement

Other data relating to the development of the CoCB tool are available by emailing AMR, anne_marie.rafferty@kcl.ac.uk.

Ethical approval

The project has been approved by the Psychiatry, Nursing and Midwifery Research Ethics Subcommittee (PNM RESC) at King’s College London (project no. PNM/13/14-153) and relevant Research & Development (R&D) departments of participating organizations.

For peer review only - http://bmjopen.bmj.com/site/about/guidelines.xhtml

References

1 Francis R. Report of the Mid Staffordshire NHS Foundation Trust public inquiry. London: The Stationery Office 2013.

2 Kirkup B. The report of the Morecambe Bay investigation. London: The Stationery Office 2015.

3 Walshe K, Shortell S. When Things Go Wrong: How Health Care Organizations Deal With Major Failures. *Health Affairs* 2004;23:103-111. doi:10.1377/hlthaff.23.3.103

4 West M A, Lyubovnikova J, Eckert R et al. Collective leadership for cultures of high quality health care. *Journal of Organizational Effectiveness: People and Performance* 2014;1:240-260. doi:10.1108/joepp-07-2014-0039

5 Care Quality Commission (CQC) Time to listen in NHS hospitals. Dignity and nutrition inspection programme 2012. Care Qulaity Comission, Newcastle Upon Tyne 2013.

6 Care Quality Commission (CQC) *Dignity and Nutrition inspection programme.* Newcastle Upon Tyne: Care Quality Commission 2011.

7 Dixon-Woods M, Baker R, Charles K et al. Culture and behaviour in the English National Health Service: overview of lessons from a large multimethod study. *BMJ Quality & Safety* 2013;23:106-115. doi:10.1136/bmjqs-2013-001947

8 Care Quality Commission (CQC) The state of health care and adult social care in England 2015/16. Newcastle Upon Tyne: Care Quality Commission 2016.

9 Dawson J. Staff experience and patient outcomes: what do we know? London: NHS Employers 2014.

10 Ghaziani A. An “amorphous mist”? The problem of measurement in the study of culture. *Theory and Society* 2009;38:581-612. doi:10.1007/s11186-009-9096-2

11 Schein E. *Organizational culture and leadership*. 4th ed. San Francisco: Jossey-Bass 2010.

12 Bushe G, Marshak R. Revisioning Organization Development: Diagnostic and Dialogic Premises and Patterns of Practice. *The Journal of Applied Behavioral Science* 2009;45:348-368. doi:10.1177/0021886309335070

13 Mannion R, Konteh F, Davies H. Assessing organisational culture for quality and safety improvement: a national survey of tools and tool use. *Quality and Safety in Health Care* 2009;18:153-156. doi:10.1136/qshc.2007.024075

14 Hesselink G, Kuis E, Pijnenburg M et al. Measuring a caring culture in hospitals: a systematic review of instruments. *BMJ Open* 2013;3:e003416. doi:10.1136/bmjopen-2013-003416

15 Francis R. Freedom to Speak Up; An independent review into creating an open and honest reporting culture in the NHS. UK Government Web Archive 2015.

16 Hinkin T. A Brief Tutorial on the Development of Measures for Use in Survey Questionnaires. *Organizational Research Methods* 1998;1:104-121. doi:10.1177/109442819800100106

17 Hinkin T. A Review of Scale Development Practices in the Study of Organizations. *Journal of Management* 1995;21:967-988. doi:10.1177/014920639502100509

For peer review only - http://bmjopen.bmj.com/site/about/guidelines.xhtml

18 Hinkin T, Tracey JB, Enz CA Scale construction: Developing reliable and valid measurement instruments. Journal of Hospitality & Tourism Research 1997; 21:100-120. doi:10.1177/109634809702100108

19 De Vaus D. *Surveys in social research*. 5th ed. London: Routledge 2002.

20 Patterson M, Nolan M, Rick J et al. *From Metrics to Meaning: Culture Change and Quality of Acute Hospital. Report for the National Institute for Health Research Service Delivery and Organisation programme.* Queen's Printer and Controller of HMSO 2011.

21 Davies H, Nutley S, Mannion R. Organisational culture and quality of health care. *Quality in Health Care* 2000;9:111-119. doi:10.1136/qhc.9.2.111

22 The King's Fund (King's Fund) Culture and Leadership in the NHS. The King's Fund 2014 Survey. London: The King's Fund 2014.

23 West M, Eckert R, Steward K et al. Developing collective leadership for health care. London: The King's Fund 2014.

24 Mannion R, Davies H, Konteh F et al. *Measuring and Assessing Organisational Culture in the NHS (OC1). Research Report Produced for the National Co-ordinating Centre for the National Institute for Health Research Service Delivery and Organisation Programme (NCCSDO).* York: The Centre for Health and Public Services Management 2008.

25 Scott T, Mannion R, Davies H et al. The Quantitative Measurement of Organizational Culture in Health Care: A Review of the Available Instruments. *Health Services Research* 2003;38:923-945. doi:10.1111/1475-6773.00154

26 NHS England. Friends and Family Test. England.nhs.uk. 2013. https://www.england.nhs.uk/ourwork/pe/fft/ (accessed 13 Feb 2017).

27 Rafferty AM, Philippou J, Fitzpatrick JM et al. Culture of Care Barometer. Report to NHS England on the development and validation of an instrument to measure 'Culture of Care' in NHS Trusts. London: National Nursing Research Unit 2015.

28 Moore G, Benbasat I. Development of an Instrument to Measure the Perceptions of Adopting an Information Technology Innovation. *Information Systems Research* 1991;2:192-222. doi:10.1287/isre.2.3.192

29 Hinkin T. A Brief Tutorial on the Development of Measures for Use in Survey Questionnaires. *Organizational Research Methods* 1998;1:104-121. doi:10.1177/109442819800100106

30 National Health Service (NHS) Five Year Forward View. NHS England 2014. https://www.england.nhs.uk/wp-content/uploads/2014/10/5yfv-web.pdf (accessed 13 Feb 2017).

31 West M, Dawson J, Admasachew L et al. NHS Staff Management and Health Service Quality. Results from the NHS Staff Survey and Related Data. Department of Health 2011. https://www.gov.uk/government/publications/nhs-staff-management-and-health-service-quality (accessed 13 Feb 2017).

For peer review only - http://bmjopen.bmj.com/site/about/guidelines.xhtml

For peer review only

For peer review only - http://bmjopen.bmj.com/site/about/guidelines.xhtml

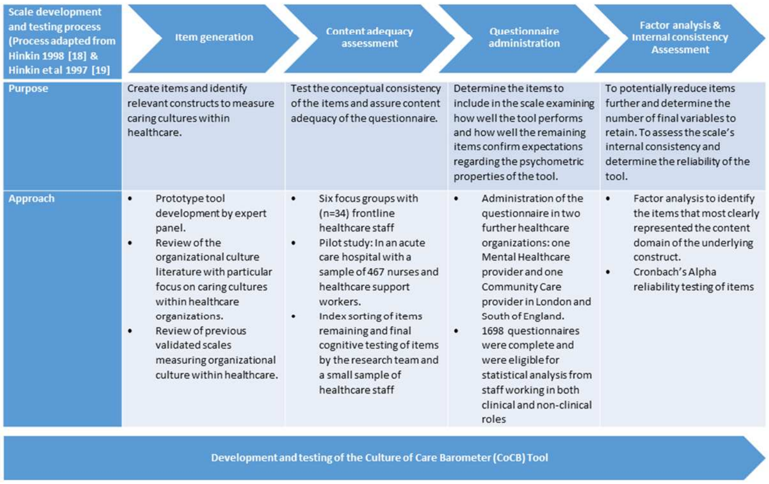

Figure 1: Process of developing and testing the Culture of Care Barometer (CoCB) tool

338x190mm (96 x 96 DPI)

review only

BMJ Open

Development and testing of the 'Culture of Care Barometer' (CoCB) in healthcare organisations: a mixed-method study.

Journal:	*BMJ Open*
Manuscript ID	bmjopen-2017-016677.R1
Article Type:	Research
Date Submitted by the Author:	06-Jun-2017
Complete List of Authors:	Rafferty, Anne Marie; King's College London, Florence Nightingale School of Nursing and Midwifery<br>Philippou, Julia; King's College London, Nursing & Midwifery<br>Fitzpatrick, Joanne; King's College London, Nursing & Midwifery<br>Pike, Geoff; Employment Research<br>Ball, Jane; National Institute for Health Research Collaboration for Leadership in Applied Health Research and Care (Wessex),University of Southampton, Centre for Innovation and Leadership in Health Sciences
**Primary Subject Heading**:	Nursing
Secondary Subject Heading:	Health services research
Keywords:	Culture of Care, tool development, Organisational development < HEALTH SERVICES ADMINISTRATION & MANAGEMENT, quality in healthcare, Human resource management < HEALTH SERVICES ADMINISTRATION & MANAGEMENT

SCHOLARONE™
Manuscripts

only

Title page

Development and testing of the 'Culture of Care Barometer' (CoCB) in healthcare organisations: a mixed-method study.

Full name, postal address, e-mail and telephone number of the corresponding author:

Professor Anne Marie Rafferty

57 Waterloo Road

London SE1 8WA

Email: anne_marie.rafferty@kcl.ac.uk

Tel: 02078483012/3984

Full name, department, institution, city and country of all co-authors:

Professor Anne Marie Rafferty, Florence Nightingale Faculty of Nursing and Midwifery, King's College London, London UK.

Dr Julia Philippou, Florence Nightingale Faculty of Nursing and Midwifery, King's College London, London UK.

Dr Joanne M. Fitzpatrick, Florence Nightingale Faculty of Nursing and Midwifery, King's College London, London UK.

Geoff Pike, Employment Research, Hove, UK.

Dr Jane Ball, Faculty of Health Sciences, University of Southampton, Southampton UK

Word count, excluding title page, abstract, references, figures and tables: 4249

For peer review only

Abstract

Objective: Concerns about care quality have prompted calls to create workplace cultures conducive to high quality, safe and compassionate care and provide a supportive environment in which staff can operate effectively. How healthcare organisations assess their culture of care is an important first step in creating such cultures. This paper reports on the development and validation of a tool, the Culture of Care Barometer, designed to assess perceptions of a caring culture among healthcare workers preliminary to culture change.

Design/Setting/Participants: An exploratory mixed methods study designed to develop and test the validity of a tool to measure 'culture of care' through focus groups and questionnaires. Questionnaire development was facilitated through; a literature review, experts generating items of interest and focus group discussions with healthcare staff across specialities, roles and seniority within three types of public healthcare organisations in the UK. The tool was designed to be multi-professional and pilot tested with a sample of 467 nurses and healthcare support workers in acute care and then validated with a sample of 1698 staff working across acute, mental health and community services in England. Exploratory factor analysis was used to identify dimensions underlying the Barometer.

Results: Psychometric testing resulted in the development of a 30-item questionnaire linked to four domains with retained items loading to four factors: organisational values (α=0.93, valid n=1568, M=3.7); team support (α=0.93, valid n=1557, M=3.2); relationships with colleagues (α=0.84, valid n=1617, M=4.0) and job constraints (α=0.70, valid n=1616, M=3.3).

Conclusions: The study developed a valid and reliable instrument with which to gauge the different attributes of care culture perceived by healthcare staff with potential for organisational benchmarking.

Strengths and limitations of the study

- This study explored the reliability, content validity and factorial structure of the Culture of Care Barometer (CoCB) tool.
- The tool was tested with a total sample of over 2000 healthcare staff.
- The tool was only tested in public healthcare settings in England and hence requires further validation across different care settings to evaluate its usefulness and acceptability with wider populations.
- The CoCB requires further validation to ascertain its concurrent and predictive validity.

For peer review only

Main text

BACKGROUND

The importance of culture in providing high quality and safe care to patients has been emphasized in many investigations of failings in healthcare systems both nationally [1, 2] and internationally [3]. Healthcare organisations have begun to look critically at ways that can improve their culture and consequently the care provided to patients [4]. In the United Kingdom (UK), the Care Quality Commission, has drawn attention to "cultures of care that are too often 'task-based' when they should be person-centred, and where the unacceptable become the norm" [5](pg.5) noting variation in cultures within organisations reflecting leadership and management failings [6]. Learning from high profile failures in care delivery indicates that quality and culture are not uniform within, let alone across, organisations [6, 7]. This was evident in the description and analysis of events (and the context to those events) at Mid Staffordshire National Health Service (NHS) Trust, described by the Robert Francis Inquiry [1]. Pockets of excellence can coexist alongside the worst examples of care failures [6]; lack of consistency in care culture impedes the spread of good practice across organisations [1, 8]. Establishing cultures that will allow healthcare organisations to achieve the ultimate goal of providing high quality care has therefore become a major policy concern.

Evidence suggests that major failures are frequently not brought to light by the systems for quality assurance or improvement that are part of most healthcare organisations in developed countries - such as incidence reporting, mortality and morbidity reviews, inspections, accreditations, clinical profiling and risk and claim management [3]. Since these cultural attributes are not picked up in the measures of quality and performance currently in use; metrics fail to capture the meaning and reality of a culture of care for patients or staff. Moreover, research in the UK demonstrates that the wellbeing of staff is closely linked to the wellbeing of patients, and staff engagement is a key predictor of a wide range of outcomes in healthcare organisations [9].

One of the first questions organisations have to consider in trying to establish such care cultures is how to assess the organisational culture. The first difficulty in this is that the concept of culture is broad and multi-faceted [10]. While culture as a concept is widely used, the term itself has been described as an 'indescribable mist' [10]. Conceptual debates over how culture is defined continue, and consequently impact how it is studied [10]. Much of the literature in healthcare favours the concept of culture as shared beliefs, norms and routines through which a society can be interpreted and understood [11]. With this definition in mind our focus was on understanding the culture of care, as a subset of organisational culture, and helping organisations gauge the different attributes of caring environments.

From an organisational development (OD) perspective - the practical application of culture assessment tools speaks to the diagnostic premises of OD theory and practices. Such tools ascertain the strengths and weaknesses of organisations and help them prescribe interventions or 'treatments' of change based on an objective diagnosis from the data collected [12]. Within healthcare there is a plethora of well-known instruments for measuring the culture of organisations and 'patient safety' culture [13]. A national survey of healthcare organisations in the UK to identify the culture assessment tools that are used within the English NHS, concluded that while organisations are increasingly using culture assessment instruments these focus primarily on the assessment of safety culture rather than perspectives of quality [13]. Moreover, while the centrality of patients' experiences, of safety, caring and supportive cultures, in such tools is well evidenced, a large research programme examining culture and behaviour in the English NHS concluded that

For peer review only

creating caring cultures where staff can feel supported, respected, valued and engaged are equally important for providing high quality care [7]. Therefore, it is argued that achieving the optimal care culture is only possible in organisations where staff feel valued, respected and supported, and when relationships are good between managers, staff, teams, departments and across institutional boundaries [7, 9, 10]. An initial analysis of the literature revealed a lack of instruments for measuring 'care cultures' from the perspective of service providers as distinct from organisational culture or patient safety culture [14].

Diagnostic OD entails a problem-based approach where organisations are considered to be problematic and need fixing [15]. This approach to data gathering has been described as 'problem-sensing' [7] as it actively seeks out weaknesses in organisational systems. This can result in organisational members being wary as they may feel that the main purpose of data collection is to attribute blame; ultimately this can inhibit or make members more resistant to change [7, 12]. One of the most sensitive messages coming out from the Mid-Staffordshire Inquiry was how staff suffered as a result of raising concerns [1]. This has led to a review of the way NHS organisations deal with concerns raised by NHS staff, advocating for a culture of safety and learning in which staff feel safe to raise concerns and these conversations take place as part of everyday practice without fear of blame or recrimination [15]. Previous research identified that extant culture assessment tools failed to address culture attributes that promote the development of a blame-free environment [13].

In contrast to diagnostic OD approaches, dialogic approaches are primarily concerned with 'meaning-making' [12]. Building a more dialogical approach to OD [12] therefore could encourage reflection and stimulate discussion about the culture of an organisation and how 'care givers' express and create meaning in their performance of care [14]. Given the gaps in current culture measurement tools we aimed to develop a tool that could act as a 'diagnostic' measurement to help organisations assess the culture of care but also as a 'dialogic' tool designed to prompt reflection on the underlying issues involved in creating a caring culture. The current paper presents the development and testing of the Culture of Care Barometer (CoCB) as a tool that has the potential to serve these purposes.

METHODS

We followed well-recognized and comprehensive approaches for instrument development and testing e.g. Hinkin's framework for scale development [16, 17, 18, 19], and pursued a variety of data collection and analysis methods to operationalize the elements that are important in creating caring cultures and to ensure the reliability of the tool [20]. A detailed account of the process of developing and testing the CoCB is provided below. Figure one provides an overview and graphical representation of this process.

Insert Figure 1 Here

Item generation

We used a mixed method approach in the creation of items to assess the culture of care construct. Our processes involved both inductive and deductive approaches to generate items [17, 18, 16]. Initially an inductive approach was used where an expert panel of six healthcare leaders developed a prototype questionnaire by generating items and domains they considered important in improving patient care. The expert panel consisted of individuals with extensive experience and expertise in

For peer review only - http://bmjopen.bmj.com/site/about/guidelines.xhtml

regulation, leadership, healthcare-delivery, management, policy and research within the English NHS, including the use of tools used in inspection regimes. This process was complemented by a deductive approach involving a comprehensive literature review in four major healthcare related electronic database resources (CINAHL, EMBASE, MEDLINE, and Web of Science; from 1945 to 2015) using key terms such as 'organisational culture' 'assessment tools', 'healthcare settings' and 'quality of care'. Through the literature review key studies [e.g. [7, 21, 22, 23, 24]] and previous comprehensive reviews on validated scales measuring organisational culture [e.g. [14, 13, 25, 26]] within healthcare were extracted and together, these two complementary processes helped us create an initial list of candidate items and facilitated the refinement of items and elaboration of domains that could be used to measure the concept of the culture of care.

Content adequacy assessment

A comprehensive approach was also followed at the stage of content adequacy to test the conceptual consistency of the items and assure content validity of the questionnaire. In order to achieve this we undertook six focus groups with a cross section of healthcare staff (N=34) of different levels of seniority and from different settings e.g. both in-patient and out-patient. The aim of these focus groups was to explore perceptions of terminology and cognitively test items and their meaning to enable a 'co-creation approach' to the development of the tool with frontline staff. This was envisaged as a process that would allow the tool development stage to address some limitations reported in the literature in terms of developing tools that are 'fit for purpose' in the NHS context. The process also allowed us to explore staff ideas of what constitutes a 'good culture of care' and key 'signs and symptoms' of an organisation that has a good or poor culture of care and identify any additional items relevant to the concept. At the same time we were able to test the appropriateness, comprehension and clarity of items, and ease and acceptability of instructions and format of the initial questionnaire [20].

At this stage an initial tool of 37 items clustered under four domains was developed and a five point scale, ranging from not at all, to fully agree, was used to record participants' agreement with the items. The first domain comprised six items that explored issues surrounding resources and quality of care, facilities and equipment, staffing levels and views of workplace in terms of safety and quality. It included the Friends and Family Test as a reference item [27] and a question about action required to improve resources. The second domain comprised 10 items relating to management and support. Ten items in the third domain addressed development, staff involvement in decision making and overall culture in the organisation. The fourth domain included 11 items about staff meetings, teamwork and feedback and willingness of the organisation to learn from issues raised as well as incidents. For each domain a question was included about how much influence participants had to improve things, rated on a five point sale (from 'none', to 'a lot'). The initial tool is provided in full in the study's report [28].

The tool was then pilot tested in an acute NHS organization with a sample of registered nurses and midwives and healthcare support workers (n=467, 24% response rate). The aim of this pilot testing was to examine how well the tool performed and test the face validity and internal reliability of the items before wider testing. An initial factor analysis was conducted to reduce the number of items and further refine the tool. Results from this pilot study are reported in the study's report [28]. The items identified through this analysis were explored by the research team using an index-card-sorting exercise [29]. This involved asking the members of the research team to sort the items into categories. The items were printed separately on a small index card each member of the team sorting the cards into groups. Each then described what they saw as the common theme relating to

For peer review only - http://bmjopen.bmj.com/site/about/guidelines.xhtml

the cards in each group. Through this analysis and process, 30 items were retained in the questionnaire representing the concept of culture of care and these were classified under seven categories: engagement, empowerment, management and leadership, values, roles, resources and team. Participants were asked to rate the extent to which they agreed with each statement on a 5-point Likert scale (from strongly disagree to strongly agree).Before administering the final questionnaire to a wider sample a small number of NHS staff reviewed the items and the overall questionnaire to identify whether this provided an accurate representation of the overall culture in their workplace. The revised version of the tool is available in full in the study report [28].

Questionnaire administration

The retained items and revised tool was administered to a wider sample with the objective of examining how well the tool performed and how well the remaining items confirmed expectations of the psychometric properties of the tool. Below we present the main procedures we followed during this stage of development and testing the CoCB tool.

Administration of the questionnaire was undertaken in two further healthcare organisations. One was a mixed mental health care provider in London with community care and in-patient beds and the other was a predominately community healthcare organisation in a mixed urban/rural setting in the South of England. A total sample approach was adopted and an electronic version of the questionnaire was sent to the two organisations for distribution. We also forwarded 1500 paper copy questionnaires for each organisation to distribute to participants with less access to the electronic version. Questionnaires were distributed in June 2014 and data collection lasted for about eight weeks.

Factor analysis and internal consistency assessment of the tool

Data were coded and entered into a Microsoft Excel spreadsheet before being exported and analysed through IBM SPSS v21 statistical software. The demographic and employment characteristics of the participants were analysed descriptively and presented as numbers and percentages. As the measurement model might differ between types of organisations (e.g. acute, community and mental health) and the type of sample (e.g. clinical and non-clinical staff); an exploratory factor analysis was undertaken to identify whether the correlations between groups of observed items originated from one or more latent variables/factors in the data. Internal consistency was tested using Cronbach's coefficient α. An Alpha reliability score above 0.7 is considered good [19, 29] therefore the target level of reliability was set at 0.70.

RESULTS

Sample characteristics

A total of 1705 staff working across mental health and community services in both clinical and non-clinical roles returned the questionnaires, organisation 1 (n=700, 25% response rate); organisation 2 (n=1005, 24% response rate), of which 1698 were found complete and eligible for statistical analysis. Table one summarise the main profile and employment characteristics of participants. Overall four-in-five (82%, n=1237) respondents were female, with a large proportion of participants indicating being age 40 or older (Table 1). Almost all sample participants (93%, n=1459) spoke English as their first or main language. There were no difference in the demographic variable by organisation. Two-

For peer review only - http://bmjopen.bmj.com/site/about/guidelines.xhtml

thirds of staff across the two organisations worked full-time (68%, n=1074). By staff group there was a similar response profile within the two organizations and overall a larger proportion of nurses responded to the questionnaire compared to other groups of staff.

Table 1: Characteristics of participants

	Percentage (n)		
	Organisation 1 (n=700)	Organisation 2 (n=1005)	Overall (n=1705)
**Female**	**75**	**87**	**82**
**English as a first or main language**	**86**	**98**	**93**
**Age groups**			
<29	8	8	8
30-39	17	17	17
40-49	33	32	32
50-59	32	35	34
60 plus	9	8	8
**Staff group**			
Registered Nurses and Health Visiting Staff	30	34	32
Healthcare Assistant/Support Worker	13	5	8
Allied Health Professionals	25	25	25
Estates and facilities	2	4	3
Doctor/Dentist	5	3	4
Administrative and Clerical Staff	15	15	15
Central Functions and Corporate Services	4	6	5
Other	4	6	5
**Work setting**			
Community	36	45	41
Clinics/Outpatient Departments	13	14	14
Wards/Inpatient Units	27	7	15
Office	16	27	23
Other	9	7	7
**Employment status**			
Full-time	76	64	68
Part-time	25	36	32

For peer review only

Structure of the tool

A factor analysis was performed on the 30 items to identify patterns of loading and extract underlying factors. Through this analysis four factors were identified. The composition of the factors was based on aggregating the scores for each item to create a single score for each factor and dividing this figure by the number of items in the CoCB tool to provide a meaningful average score. The relevant loading of items for each of the four factors is presented in Table 2.

The 12 items of the first sub scale were predominately related to the macro level elements within organisations that influence culture such as valuing employees, good communication within the organisation and visible leadership at the top level. Therefore, this subscale was considered to address wider 'organisational values'. Eleven items were loading to the second factor labelled as Team support. Items loading to this subscale were concerned with the 'meso level' of organisations

and included elements that described primarily team support relationships and management and development of employees within organisations. The remaining seven items concerned aspects of every day work at the micro level within organisations and these were loading to two factors. One sub-scale was mainly associated with four items describing social elements of work such as respect and social support between co-workers and the final three items were concerned with the ability of employees to do their job within the limits of time and resources available to them (Factor 4).

Table 2: Factor analysis and loading of items

**Subscales and items**	**Loading**
**Factor 1: Organisational Values (Macro Level)**	
The Trust listens to staff views	0.84
The Trust has a positive culture	0.77
There is strong leadership at the highest level in the Trust	0.75
I am able to influence how things are done in the Trust	0.74
I would recommend this Trust as a good place to work	0.70
I feel well informed about what is happening in the Trust	0.70
Staff successes are celebrated by the Trust	0.70
The Trust values the service we provide	0.69
Trust managers know how things really are	0.68
I am proud to work in this Trust	0.65
A positive culture is visible where I work	0.50
I get the training and development I need	0.40
**Factor 2: Team Support (Meso Level)**	
I feel well supported by my line manager	0.87
My line manager treats me with respect	0.84
My line manager gives me constructive feedback	0.83
My concerns are taken seriously by my line manager	0.81
I feel part of a well-managed team	0.60
I am kept well informed about what is going on in our team	0.52
I feel supported to develop my potential	0.50
I know who my line manager is	0.45
I am able to influence the way things are done in my team	0.44
I feel able to ask for help when I need it	0.44
Unacceptable behaviour is consistently tackled	0.40
**Factor 3: Relationships with colleagues (Micro Level)**	
The people I work with are friendly	0.81
When things get difficult, I can rely on my colleagues	0.79
I feel respected by my co-workers	0.76
I have positive role models where I work	0.56
**Factor 4: Job constrains (Micro Level)**	
I have sufficient time to do my job well	0.79
I have the resources I need to do a good job	0.72
I know exactly what is expected of me in my job	0.41

Reliability analyses were performed on each factor to identify how items loading to factors were considered a positive endorsement of the sub-scale. The results of these analyses are presented in Table 3. Cronbach's α for the macro level (Factor 1: Organisational values) and meso level scales (Factor 2: Team support) were very high both at 0.93. Cronbach's α for the micro level scales were: 0.84 (Factor 3: Relationships with colleagues) and 0.70 (Factor 4: Job constraints).

For peer review only - http://bmjopen.bmj.com/site/about/guidelines.xhtml

Table 3: Scale characteristics (Cronbach's coefficient α reliability analysis)

	**Factor 1** Organisational values (Macro Level)	**Factor 2** Team support (Meso Level)	**Factor 3** Relationship with Colleagues (Micro Level)	**Factor 4** Job constrains (Micro Level)
Total Items loading	12	11	4	3
Alpha reliability	0.93	0.93	0.84	0.70
Number of valid responses	1568	1557	1617	1616
Mean score	3.7	3.2	4.0	3.3

Both sorting and factor analytical techniques were used to assess the content adequacy of the 30 items. The initial sorting of the items undertaken as part of the content adequacy process identified seven themes. The exploratory factor analysis did not confirm the distinction among the seven themes and instead the results yielded a four factor solution with factors indicating greater emphasis for organisational values and team and social support compared to job constraints. While this process of developing the tool provides confidence in the four factors identified, confirmatory factor analysis with another independent sample will provide a more rigorous test of the loading of items.

Usefulness and added value of the tool to healthcare organisations

The results from the CoCB were presented to the participating healthcare organisations through two independent reports detailing the findings from testing the tool in each organisation. Following the presentation of the results we invited key individuals from each organisation (n=5) to a follow up discussion to receive feedback on the usefulness of the tool and explore whether the tool met the dialogic and diagnostic premises of organisational development [12]. These individuals held strategic leadership positions within the two organisations with responsibilities for overseeing workforce and culture initiatives. These sessions were audio recorded and analysed thematically.

From a diagnostic perspective the consensus overall was that the CoCB tool resonated with other instruments used in the organisations, adding 'colour and depth' to them.

> *"I think it is a much richer type of feedback than we get from the staff survey. We liked the logic and flow and could appreciate the sense of questions." (Organisational Development Manager, Organisation 2)*

> *"…you get these action plans that come out of the staff surveys, but the detail is not the depth of information that we had with this, so this enables you to think more about why, then, and question, as oppose to, 'oh, right, we've got to do something on that area." (Senior Manager, Organisation 1)*

For peer review only - http://bmjopen.bmj.com/site/about/guidelines.xhtml

The brevity of the tool, the fact that it was easy to complete and was perceived as targeting the right domains was appreciated by staff. Moreover, from a diagnostic perspective the tool was perceived by participants as useful in providing a reference point for them to gauge where they were on a cultural spectrum or journey.

From a dialogical perspective the fundamental value of the CoCB tool was reflected in the belief that 'culture changes by talking about it' and the Barometer helped to surface issues for discussion. Data from the CoCB were seen as helpful in drilling into further detail or using it as a prompt for a 'quality conversation' for instance, with smaller, discrete groups, teams or where it was felt things were not quite right or when organisations felt the need to gauge the impact of changes they had made.

> *'Culture …does not change overnight and the fact that it is [the CoCB tool] a prompt to reflect upon has been I think a really powerful aspect of the tool.'(Senior Executive, Organisation 2).*

Finally, the commentary element of the tool was identified as providing a rich source of intelligence in helping to unpack notions of culture:

> *"trying to understand what it is that matters to staff and what they feel about the place that they work in" (Workforce Transformation Lead, Organisation 2).*

It was also regarded as helpful in picking up on contradictions that might exist in organisations, as one senior manager observed:

> *"where you've got high scores for 'my manager treats me with respect', but then, 'Oh, I don't think my manager understands what the real world is like' (Leadership Project Manager, Organisation 1)*.

Richer feedback via the CoCB in comparison to the staff survey helped to tease out the contradictions and take the quality of conversations at team level to the next stage.

DISCUSSION

Enabling the workforce to put the right things in place for patients is key to improving NHS performance in terms of quality and safety [30, 31] and this is the underlying principle of the CoCB as a tool. The challenge all organisations face is that there is not a one-size-fits-all solution as each individual is unique and will react differently to the challenges and values of an organisation. The CoCB can provide feedback from staff to enhance understanding of the factors that contribute to a culture lacking in care and safety. The CoCB appeared to perform well in meeting the gap in the literature which suggests that there is a need for tools to assess culture with a focus on formative diagnostic purposes to support reflexive practice [13]. The focus on the carers' views helps to comprehend the intersection of individual and organisational factors which distinguish the CoCB from other tools that prioritise either macro or micro levels. Moreover, using a dialogic approach to organisational development we created a tool that can be a resource to facilitate the involvement of frontline staff, at different levels and with different roles, in culture change initiatives.

The CoCB tool possesses several advantages over existing tools. During the development and testing of the tool we addressed concerns reported in the literature and from participants about the need for tools that are 'fit for purpose' [13] and are not onerous and time consuming to complete. One of

For peer review only - http://bmjopen.bmj.com/site/about/guidelines.xhtml

the major strengths of this study was the co-creation of the tool in collaboration with frontline staff providing care within NHS organisations. Three main sources of data and information were used to create the content of the tool namely a prototype tool developed by an expert panel in this field, a comprehensive literature review of the concept of culture of care and other tools used within healthcare, and input from interviews with staff working in healthcare organisations. Feedback from participants indicated that the data collected via the CoCB could provide extra intelligence about the jigsaw of what is happening within organisations. Participants indicated that the tool could enhance or complement current data collection methods used in hospital settings such as the Friends and Family Test and the Staff Survey to identify concerns about poor care. In this way providing a more comprehensive picture of patient and staff satisfaction with services provided. In addition, participants spoke of the struggle to make meaningful changes based on Staff Survey feedback alone and they shared that data collected via the CoCB alerted them to important contextual feedback and factors that in some cases were more useful in planning and developing action plans.

While the CoCB is a reliable tool and does seem to fill a niche for identifying and understanding some of the social processes at work within an organisation, we recognise that not all factors contributing to developing a caring culture may be included in the tool. We also acknowledge that not all the items may be relevant to all healthcare contexts and we recommend that testing and adaptation of these items may be necessary in future validations. Therefore, practitioners should use the CoCB in conjunction with other tools that can help organisations achieve culture change. Moreover, the sample of this testing was predominately nurse led, which may be a direct result of the proportion of nurse personnel working in the healthcare environments. However, cultures are co-created by all members of an organisation and this means that everyone is responsible for the welfare of the organisation as a whole. Further work is needed to test the reliability and validity of the questionnaire as well as engagement and uptake of the tool with other professional groups. While the tool has been developed in the context of the UK NHS, future work to adjust and test the tool in other organisations and countries will allow validation of the tool in a global context. Whilst the development of the tool was prompted by an interest in the contexts that may support the delivery of patient centric care, we have yet to examine if environments with more positive cultures of caring do indeed have care that is considered to be more patient centric – by staff or patients. Future work could investigate the hypothesized relationships between the culture of care and achievement of patient-centred care delivery, staff satisfaction, work-engagement and a reduction in work-related burnout as well as student learning.

Measuring and monitoring culture is a recurrent challenge in healthcare, the CoCB could act as a diagnostic, and practical tool for organisations to embrace as a first step in improvement work as well as a means of monitoring change over time. The tool has potential use in clinical practice and research. It is easy to administer, can be completed and analysed quickly providing timely feedback that can be used by organisations to identify areas of strength and weakness and help with the planning of continuous quality improvements or culture change initiatives that hospitals are undertaking. The wider applicability of the tool needs to be explored in future studies and its relevance for groups that may not have a direct role in care provision but who nevertheless are an important part of the organisation and whose feedback is important as culture is every one's business [23]. At the time of writing this paper a digital version of the tool is being developed. Further research should extend the current efforts to refine and evaluate the impact of the CoCB as necessary in order to develop interventions that can improve the culture of care in healthcare environments.

For peer review only

Twitter Follow Anne Marie Rafferty at @annemarieraffer, Julia Philippou at @julia_philippou and Jane Ball at @JaneEBall

Acknowledgments

We would like to thank all those who contributed to this work, particularly the staff who took part in in the survey and those who engaged in the focus groups. We also acknowledge the role of the Passionate About Care Today 'PACT' group (Baroness Audrey Emerton, Professor Dame Elizabeth Fradd, Professor Tricia Hart, Sir Stephen Moss, Flo Panel Coates, Professor Anne Marie Rafferty) in creating the prototype Culture of Care Barometer upon which this work is based and for their time and insights throughout the subsequent development of the tool. We are grateful to the support and insights provided by the Steering Group for the project: Caroline Alexander, Bronagh Scott, Flo Panel Coates, Yvonne Coghill, Morvia Gooden; Sylvia Tang, Virginia Minogue, Jane Clegg, Paul Taylor, Nigel Charlesworth, Hana Wild and Savaia Stevenson.

Contributors

AMR and JP are joint first authors. AMR was the principal investigator and with JB conceived and designed the study. AMR, JP, JF, GP and JB all contributed to the collection, analysis, or interpretation of data for the work. JP developed the first draft of the paper and all the authors contributed to different sections and revised the paper critically for intellectual content. All authors gave final approval of the version to be published and agree to be accountable for all aspects of the work in ensuring that question related to accuracy or integrity of any part of the work are appropriately investigated and resolved.

Funding

This work was supported by funding received from NHS London and NHS England. The independent research reported in this paper formed part of the Compassion in Practice, national strategy for nursing, midwifery and care staff, led by Jane Cummings the Chief Nursing Officer for NHS England, under action area four 'Building and Strengthening Leadership-leading with Compassion'.

Competing interests

None declared.

Data sharing statement

Other data relating to the development of the CoCB tool are available by emailing AMR, anne_marie.rafferty@kcl.ac.uk.

Ethical approval

The project has been approved by the Psychiatry, Nursing and Midwifery Research Ethics Subcommittee (PNM RESC) at King's College London (project no. PNM/13/14-153) and relevant Research & Development (R&D) departments of participating organizations.

For peer review only

References

1 Francis R. Report of the Mid Staffordshire NHS Foundation Trust public inquiry. London: The Stationery Office 2013.

2 Kirkup B. The report of the Morecambe Bay investigation. London: The Stationery Office 2015.

3 Walshe K, Shortell S. When Things Go Wrong: How Health Care Organizations Deal With Major Failures. *Health Affairs* 2004;23:103-111. doi:10.1377/hlthaff.23.3.103

4 West M A, Lyubovnikova J, Eckert R et al. Collective leadership for cultures of high quality health care. *Journal of Organizational Effectiveness: People and Performance* 2014;1:240-260. doi:10.1108/joepp-07-2014-0039

5 Care Quality Commission (CQC) Time to listen in NHS hospitals. Dignity and nutrition inspection programme 2012. Care Quality Commission, Newcastle Upon Tyne 2013.

6 Care Quality Commission (CQC) *Dignity and Nutrition inspection programme.* Newcastle Upon Tyne: Care Quality Commission 2011.

7 Dixon-Woods M, Baker R, Charles K et al. Culture and behaviour in the English National Health Service: overview of lessons from a large multimethod study. *BMJ Quality & Safety* 2013;23:106-115. doi:10.1136/bmjqs-2013-001947

8 Care Quality Commission (CQC) The state of health care and adult social care in England 2015/16. Newcastle Upon Tyne: Care Quality Commission 2016.

9 Dawson J. Staff experience and patient outcomes: what do we know? London: NHS Employers 2014.

10 Ghaziani A. An “amorphous mist”? The problem of measurement in the study of culture. *Theory and Society* 2009;38:581-612. doi:10.1007/s11186-009-9096-2

11 Schein E. *Organizational culture and leadership*. 4th ed. San Francisco: Jossey-Bass 2010.

12 Bushe G, Marshak R. Revisioning Organization Development: Diagnostic and Dialogic Premises and Patterns of Practice. *The Journal of Applied Behavioral Science* 2009;45:348-368. doi:10.1177/0021886309335070

13 Mannion R, Konteh F, Davies H. Assessing organisational culture for quality and safety improvement: a national survey of tools and tool use. *Quality and Safety in Health Care* 2009;18:153-156. doi:10.1136/qshc.2007.024075

14 Hesselink G, Kuis E, Pijnenburg M et al. Measuring a caring culture in hospitals: a systematic review of instruments. *BMJ Open* 2013;3:e003416. doi:10.1136/bmjopen-2013-003416

15 Francis R. Freedom to Speak Up; An independent review into creating an open and honest reporting culture in the NHS. UK Government Web Archive 2015.

16 Hinkin T. A Brief Tutorial on the Development of Measures for Use in Survey Questionnaires. *Organizational Research Methods* 1998;1:104-121. doi:10.1177/109442819800100106

17 Hinkin T. A Review of Scale Development Practices in the Study of Organizations. *Journal of Management* 1995;21:967-988. doi:10.1177/014920639502100509

For peer review only

18 Hinkin T, Tracey JB, Enz CA Scale construction: Developing reliable and valid measurement instruments. Journal of Hospitality & Tourism Research 1997; 21:100-120. doi:10.1177/109634809702100108

19 De Vaus D. *Surveys in social research*. 5th ed. London: Routledge 2002.

20 Patterson M, Nolan M, Rick J et al. *From Metrics to Meaning: Culture Change and Quality of Acute Hospital. Report for the National Institute for Health Research Service Delivery and Organisation programme.* Queen's Printer and Controller of HMSO 2011.

21 Davies H, Nutley S, Mannion R. Organisational culture and quality of health care. *Quality in Health Care* 2000;9:111-119. doi:10.1136/qhc.9.2.111

22 The King's Fund (King's Fund) Culture and Leadership in the NHS. The King's Fund 2014 Survey. London: The King's Fund 2014.

23 West M, Eckert R, Steward K et al. Developing collective leadership for health care. London: The King's Fund 2014.

24 Mannion R, Davies H, Konteh F et al. *Measuring and Assessing Organisational Culture in the NHS (OC1). Research Report Produced for the National Co-ordinating Centre for the National Institute for Health Research Service Delivery and Organisation Programme (NCCSDO).* York: The Centre for Health and Public Services Management 2008.

25 Scott T, Mannion R, Davies H et al. The Quantitative Measurement of Organizational Culture in Health Care: A Review of the Available Instruments. *Health Services Research* 2003;38:923-945. doi:10.1111/1475-6773.00154

26 NHS England. Friends and Family Test. England.nhs.uk. 2013. https://www.england.nhs.uk/ourwork/pe/fft/ (accessed 13 Feb 2017).

27 Rafferty AM, Philippou J, Fitzpatrick JM et al. Culture of Care Barometer. Report to NHS England on the development and validation of an instrument to measure 'Culture of Care' in NHS Trusts. London: National Nursing Research Unit 2015.

28 Moore G, Benbasat I. Development of an Instrument to Measure the Perceptions of Adopting an Information Technology Innovation. *Information Systems Research* 1991;2:192-222. doi:10.1287/isre.2.3.192

29 Hinkin T. A Brief Tutorial on the Development of Measures for Use in Survey Questionnaires. *Organizational Research Methods* 1998;1:104-121. doi:10.1177/109442819800100106

30 National Health Service (NHS) Five Year Forward View. NHS England 2014. https://www.england.nhs.uk/wp-content/uploads/2014/10/5yfv-web.pdf (accessed 13 Feb 2017).

31 West M, Dawson J, Admasachew L et al. NHS Staff Management and Health Service Quality. Results from the NHS Staff Survey and Related Data. Department of Health 2011. https://www.gov.uk/government/publications/nhs-staff-management-and-health-service-quality (accessed 13 Feb 2017).

For peer review only

Figure 1: Process of developing and testing the Culture of Care Barometer (CoCB) tool

For peer review only

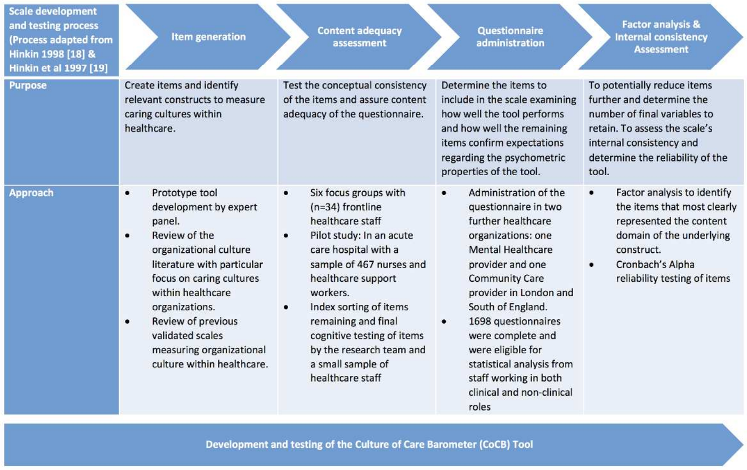

Figure 1: Process of developing and testing the Culture of Care Barometer (CoCB) tool

297x209mm (300 x 300 DPI)

iew only
